# Residual sweeping errors in turbulent particle pair diffusion in a Lagrangian diffusion model

**DOI:** 10.1371/journal.pone.0189917

**Published:** 2017-12-18

**Authors:** Nadeem A. Malik

**Affiliations:** Department of Mathematics and Statistics, King Fahd University of Petroleum and Minerals, P.O. Box 5046, Dhahran 31261, Saudi Arabia; University of Washington, UNITED STATES

## Abstract

Thomson, D. J. & Devenish, B. J. [*J. Fluid Mech*. 526, 277 (2005)] and others have suggested that sweeping effects make Lagrangian properties in Kinematic Simulations (KS), Fung et al [Fung J. C. H., Hunt J. C. R., Malik N. A. & Perkins R. J. *J. Fluid Mech*. 236, 281 (1992)], unreliable. However, such a conclusion can only be drawn under the *assumption* of locality. The major aim here is to quantify the sweeping errors in KS without assuming locality. Through a novel analysis based upon analysing pairs of particle trajectories in a frame of reference moving with the large energy containing scales of motion it is shown that the normalized integrated error $e_{K}^{I}$ in the turbulent pair diffusivity (*K*) due to the sweeping effect decreases with increasing pair separation (*σ*_*l*_), such that $e_{K}^{I}\to 0$ as *σ*_*l*_/*η* → ∞; and $e_{K}^{I}\to \infty$ as *σ*_*l*_/*η* → 0. *η* is the Kolmogorov turbulence microscale. There is an intermediate range of separations 1 < *σ*_*l*_/*η* < ∞ in which the error $e_{K}^{I}$ remains negligible. Simulations using KS shows that in the swept frame of reference, this intermediate range is large covering almost the entire inertial subrange simulated, 1 < *σ*_*l*_/*η* < 10^5^, implying that the deviation from locality observed in KS cannot be atributed to sweeping errors. This is important for pair diffusion theory and modeling. PACS numbers: 47.27.E?, 47.27.Gs, 47.27.jv, 47.27.Ak, 47.27.tb, 47.27.eb, 47.11.-j.

## 1 Introduction

Turbulent particle pair diffusion has attained somewhat of an iconic status in the turbulence community, many researchers having addressed this topic over the decades. Nevertheless, most if not all theories of turbulent particle pair diffusion in homogeneous turbulence with extended inertial ranges have been based upon the hypothesis of locality since Richardson in 1926 [1], and Obukhov in 1941 [2]. See also [3–5] for recent discussions of this topic.

Richardson pioneered this field and introduced the idea of a scale dependent pair diffusivity as the fundamental quantity of interest in turbulent pair diffusion studies. The turbulent pair diffusivity is defined as,

$$\begin{array}{c} K\left(l\right)=\frac{1}{2}\frac{d\langle l^{2}\rangle }{dt}=\left\langle l\cdot v\left(l\right)\right\rangle , \end{array}$$ (1)

where **l**(*t*) is the pair displacement vector at time *t*, *l* = |**l**|, **v**(*l*) is the pair relative velocity, and 〈⋅〉 is the ensemble average over all particle pairs.

Locality states that the further increase in separation of particle pairs which are separated at a distance of *l* in homogeneous isotropic and statistically stationary turbulence is determined only by the energies contained in the eddies of a similar size to *l*. This, leads to the scaling $K∼\langle lv\rangle ∼l\sqrt{E(l)}$, and assuming that the turbulence energy spectrum is *E*(*l*) ∼ *l*^5/3^, then *K*(*l*) ∼ *l*^4/3^ is readily obtained, showing the equivalence of locality and the 4/3 law.

In passing, we note that alternative scalings for *K* have been proposed in the past. Hentchel and Procaccia [6] proposed the more general scaling *K* ∼ *ε*^*a*^*l*^*b*^*t*^*c*^, for 2*a* + *c* = 2 and 3*a* − *b* = 1, and *c* ≠ 0 in their work on a fractal based model for cloud dispersion; see also Klafter [7]. In fact Batchelor [8] had earlier proposed that *K* = *K*(*t*) could be made dependent on the time alone—here the time is the time from release of the particle pair. However, such a time-dependent pair diffusivity would give unrealistically high values for *K*(*t*) at large times (tending to infinity). For short times, however, when the relative velocity is highly correlated with its initial value, such a scaling can recover the well known short-time ballistic motion although it lasts only for a very short time. As our interest here is in the pair diffusion scalings inside the inertial subrange when the times after release are much greater than the short ballistic time scale, we will not consider a time-dependent pair diffusivity here.

The locality hypothesis can readily be applied to generalized power law spectra of the type, *E*(*k*) ∼ *k*^−*p*^, for 1 < *p* ≤ 3; the pair diffusivity then scales like $K\left(l,p\right)∼\sigma_{l}^{\gamma_{p}^{l}}$ with $\gamma_{p}^{l}=\left(1+p\right)/2$, [9], where $\sigma_{l}^{2}=\langle l^{2}\rangle$. For Kolmogorov turbulence *p* = 5/3, this gives the well known Richardson scaling $K∼\sigma_{l}^{4/3}$, which Obukhov showed is equivalent to 〈*l*^2^〉 ∼ *t*^3^ [2].

Kinematic Simulations (KS) [10, 11] has often been used to investigate turbulent pair diffusion. KS is a kinematic Lagrangian model, closer to stochastic models rather than DNS, where the velocity is prescribed as a Fourier series from the start. It is not dynamical, but it has the advantage that it can simulate very large energy spectra which are beyond current experimental or DNS capabilities. This is useful for testing high Reynolds number scaling laws. However, KS does not yield the assumed locality scaling for *p* = 5/3, yielding instead $K∼\sigma_{l}^{1.53}$. For this reason, it has been assumed that KS must be in error.

Thomson & Devenish [12], for example, argue that the turbulent pair diffusivity must scale like,

$$\begin{array}{c} K\left(l\left(t\right)\right)∼S\left(l\right)\tau_{s}\left(l\left(t\right)\right), \end{array}$$ (2)

where *S*(*l*) is the structure function of the turbulence velocity field and *τ*_*s*_(*l*) is an effective time scale of velocity increments. In real turbulence, assuming locality scaling for *S*(*l*) ∼ *l*^2/3^ and for *τ*_*s*_(*l*) ∼ *l*^2/3^ leads to Richardson’s classical scaling $K\left(l\right)∼\sigma_{l}^{4/3}$, where we evaluate *K* at typical values of *l*, namely *σ*_*l*_ = 〈*l*^2^〉^1/2^ which is commonly assumed in these studies.

Thomson & Devenish argue that in KS because of the lack of true dynamical sweeping, the time scale must be a sweeping time scale, namely *τ*_*s*_ ∼ *l*/*U*_*s*_ which is a time scale for the large scales flow to cut through smaller local eddies, *U*_*s*_ being the sweeping velocity scale. This leads to, $K∼\sigma_{l}^{5/3}$. Even when the rms turbulence velocity *u*′ is taken instead of *U*_*s*_, they obtained $K∼\sigma_{l}^{14/9}=\sigma_{l}^{1.555}$ which is very close to that obtained in KS.

They concluded that whereas locality is true in real turbulence, it is not true in KS. In turbulence the large energy containing eddies carry the smaller eddies, but in KS as there is an absence of true dynamics the large scales force the fluid particles to cut through the smaller eddies in an unphysical manner, a view supported by Nicolleau & Nowakowski [13], and Eyink & Benveniste [14].

However, Thomson & Devenish address only the scaling laws in the diffusivity *K*, but do not quantified the errors in *K* in KS—is it large or small? This is important because although it is accepted that KS lacks true physical sweeping effects, if these errors are quantitatively small under given circumstances, then KS could still yield accurate and meaningful results under these conditions.

Furthermore, it is important to observe that the locality hypothesis itself, for the pair diffusion, has never been confirmed unequivocally as noted by Salazar & Collins [3], “… there has not been an experiment that has unequivocally confirmed R-O scaling over a broad-enough range of time and with sufficient accuracy”.

As such, in the absence of definitive proof of Richardson’s pair diffusion hypothesis, there is room for new thinking in this field, and it is reasonable to explore alternative, more analytic, approaches to address this problem, which is the main concern of this work.

Here we re-examine the sweeping effect in KS with a view of quantitfying the error in the KS pair diffusivity *K*^*s*^ compared to the physical pair diffusivity *K*. For this purpose, we focus upon the *differences* in the relative velocities along pairs of particle paths in the *sweeping frame of reference*. This frame of reference accounts for the physical sweeping effect of the largest energy containing scales; but a residual sweeping effect still remains due to the largest inertial range eddies sweeping the smaller inertial range eddies.

**Fig 1:**
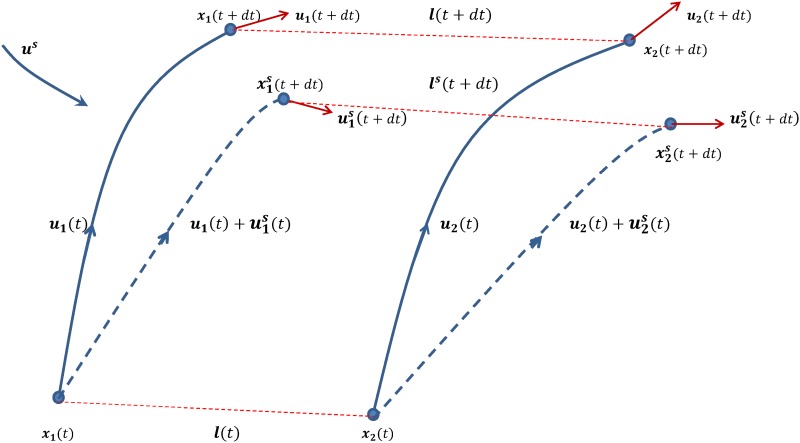
Schematic diagram illustrating the system discussed in the text. The locations of two nearby particles, labelled 1 and 2, are located at **x**_1_(*t*) and **x**_2_(*t*) respectively, with turbulence velocities **u**_1_(*t*) and **u**_2_(*t*), at time *t*; their separation is *l*(*t*) = |**x**_2_ − **x**_1_|. They are transported with velocities $\left(u_{1}+u_{1}^{s}\right)\left(t\right)$ and $\left(u_{2}+u_{2}^{s}\right)\left(t\right)$ respectively to the new locations $x_{1}^{s}\left(t\right)$ and $x_{2}^{s}\left(t\right)$ at the next time step *t* + *dt*, as shown.

Consider Fig 1 which shows a particle pair with separation **l** in the inertial subrange being swept by a large scale flow. A real fluid particle pair will be swept by the physical velocity field **u** and will follow certain particle paths; but a KS flow will transport the pair along neighbouring particle paths due to an additional KS sweeping motion, **u**^*s*^, and thereby force the particles to cut through local flow structures.

The large scale physical sweeping velocity is assumed not to affect the relative motion of particles in a pair in the inertial subrange. The critical question is, are the deviations from the physical trajectories induced by KS in the pair diffusion process large or small?

A mean flow will also sweep the smaller scales; but in a KS flow it will force a fluid particle through smaller flow structures very quickly. Thus KS clearly is not suitable for particle diffusion in the presence a non-zero mean flow. Therefore, in the ensuing analysis the error between the KS and the physical pair diffusivities, |*K*^*s*^ − *K*|, will be calculated assuming zero mean flow. We will consider generalised power law energy spectra, *E*(*k*) ∼ *k*^−*p*^, because the analysis is valid for all such power spectra and this will add weight to the conclusions that can be drawn from this work if validated over the whole range of *p* considered.

In this paper we address only the scalings and the quantitative estimates for the sweeping errors in KS. The main questions of interest are, is the KS sweeping error large or small, and in what range of separations? These questions are addressed first through a novel mathematical analysis focussing upon pairs of neighbouring particle trajectories. This is then verified against simulations using KS with very large inertial subranges.

In Section 2, we derive an expression for the error in the pair diffusivity in KS flows by analysing neighbouring trajectories in the swept frame of reference. In Section 3, the KS method is discussed and simulation results presented. In the final Section 4, we discuss the results and its implications for theory and modeling.

## 2 The normalized error in the pair diffusivity

### 2.1 The numerical timestep error

The idea of sweeping scales of motion implies a clear separation of scales between the sweeping and the swept scales. In reality, although there is a broad division between large and small scales, there is no well-defined cut-off between them because the spectrum of turbulence is continuous.

However, the situation in KS is easier because all scales are already separated by construction as a Fourier series, Eq (27), so it is a trivial matter to ‘remove’ scales of motion simply by eliminating those terms in the Fourier series. In the present case, the large sweeping scales are approximated as those scales where *k* < *k*_1_. Thus, in the swept frame of reference the sweeping action itself can be approximatedd in KS by setting *E*(*k*) = 0 for *k* < *k*_1_. However, there still remains a residual sweeping caused by the largest of the inertial scales sweeping the scales local to the pair separation.

In the ensuing analysis we will make use of Taylor expansions applied to KS flow fields. It is important to note that this is possible because although KS is a Lagrangian method, it is not in the genre of stochastic models such as Random Walk models. In KS, we generate an ensemble of flow fields, {**u**^*n*^}, *n* = 1, 2, 3, … in which each flow field **u**^**n**^ is a Fourier series with given coefficients and is therefore smooth and differentiable. The randomness in KS comes from choosing each Fourier mode and each coefficient in the Fourier expansion in each flow field **u**^**n**^ randomly from specified probability distributions, the square of the magnitudes of the coefficients being proportional to a given energy spectrum, see Section 3.2.

Consider an ensemble of particle pairs released in a field of homogeneous turbulence at time *t* = 0 with some small initial separation *l*_0_. At some time *t* later, the ensemble average of the separation is assumed to be well inside the inertial subrange and the relative motions are independent of *l*_0_ [8].

Consider the particles in one of these pairs, labeled 1 and 2, as shown in Fig 1. The particle locations are **x**_1_(*t*) and **x**_2_(*t*) respectively at time *t*; and the pair displacement is **l**(*t*) = **x**_2_ − **x**_1_, and *l*(*t*) = |**x**_2_ − **x**_1_|. **u**(**x**, *t*) is the flow due to inertial range of turbulent motions that are effective in increasing the pair separation. We will refer to **u**(**x**, *t*) as the ‘physical’ flow. All quantities are assumed at time *t* unless otherwise stated.

At time *t* the *additional* (or *residual*) KS sweeping flow **u**^*s*^(**x**, *t*) is ‘switched on’—this is not to be confused with the total KS velocity field which is $\tilde{u}=\left(u+u^{s}\right)\left(x,t\right)$, see Fig 1. In a real flow field, the small scale turbulence would be carried along dynamically by the larger scales, and therefore we could assume **u**^*s*^(**x**, *t*) = 0. In KS, the larger scales force a particle to cut through the smaller scales, hence **u**^*s*^(**x**, *t*) ≠ 0; but the spatial gradients of **u**^*s*^ are much smaller than the spatial gradients of **u**.

The flow **u**(**x**, *t*) transports the particles to **x**_1_(t*) and **x**_2_(t*) respectively at the next time step t* = *t* + *dt*; while the KS flow (**u** + **u**^*s*^)(**x**, *t*) transports the particles to $x_{1}^{s}\left(t^{*}\right)$ and $x_{2}^{s}\left(t^{*}\right)$ respectively. Note that $l^{s}=x_{2}^{s}-x_{1}^{s}$, and $l^{s}=\left|x_{2}^{s}-x_{1}^{s}\right|$.

The superscript * will refer to quantities at time t*, e.g. l* = *l*(*t* + *dt*). The superscript ^*s*^ will refer to quantities related to the KS residual sweeping, e.g. *l*^*s*^(t*) = *l*^*s*^(*t* + *dt*).

The following quantities are defined:

**u** = **u**(**x**, *t*) is the ‘physical’ fluid velocity field
**u**^*s*^ = **u**^*s*^(**x**, *t*) is the additional (residual) sweeping velocity field
**v**(**l**) = **u**(**x**_2_) − **u**(**x**_1_) is the ‘physical’ relative velocity
**v**^*s*^(**l**) = **u**^*s*^(**x**_2_) − **u**^*s*^(**x**_1_) is the additional (residual) relative velocity
$\tilde{u}=\left(u+u^{s}\right)\left(x,t\right)$ is the total KS velocity
$\tilde{v}\left(l^{s}\right)=v\left(l^{s}\right)+v^{s}\left(l^{s}\right)$ is the total KS relative velocity

We will simplifying the notation as much as possible, e.g. **u**_2_ = **u**(**x**_2_, *t*), and $u_{2}^{*}=u\left(x_{2},t+dt\right)$.

In the following analysis, we consider the situation where particle pairs have been released at some earlier time, and now at time *t* later, the pair separation *l* = |**l**| is still small compared to some characteristic large length scale *L* and inside the inertial subrange, i.e. *η* ≪ *l* ≪ *L*; where *η* is the Kolmogorov scale. we will assume that inertial subrange is large enough, *L*/*η* ≫ 1, such that inertial range scaling will apply.

Furthermore, the following Taylor expansions in the KS velocity fields will be assumed,

$$x_{1}^{s}\left(t^{*}\right)=x_{1}\left(t^{*}\right)+u_{1}^{s}\left(t\right)dt$$ (3)

$$x_{2}^{s}\left(t^{*}\right)=x_{2}\left(t^{*}\right)+u_{2}^{s}\left(t\right)dt$$ (4)

$$\begin{array}{ccc} l^{s*} & = & x_{2}^{s}\left(t^{*}\right)-x_{1}^{s}\left(t^{*}\right)\\ & = & l\left(t^{*}\right)+\left(u_{2}^{s}-u_{1}^{s}\right)dt\\ & = & l^{*}+v^{s}\left(l\right)dt \end{array}$$ (5)

to leading order in *dt*, where the increment, *dt*, is small compared to all other timescales in the system, governed only by the need for numerical accuracy.

$\tilde{v}\left(l^{s*}\right)$ is calculated at the new KS swept particle locations. Using Taylor expansions wherever necessary, assuming that the velocity fields are at least twice differentiable in space and at least once in time,

$$\begin{array}{ccc} \tilde{v}\left(l^{s*}\right) & = & \left(u+u^{s}\right)\left(x_{2}^{s}\left(t^{*}\right)\right)-\left(u+u^{s}\right)\left(x_{1}^{s}\left(t^{*}\right)\right)\\ & = & u\left(x_{2}^{s}\left(t^{*}\right)\right)-u\left(x_{1}^{s}\left(t^{*}\right)\right)+u^{s}\left(x_{2}^{s}\left(t^{*}\right)\right)-u^{s}\left(x_{1}^{s}\left(t^{*}\right)\right)\\ & = & v\left(l^{*}\right)+v^{s}\left(l^{*}\right)+\\ & & (u_{2}^{s}\cdot \nabla u_{2}\left(t^{*}\right)-u_{1}^{s}\cdot \nabla u_{1}\left(t^{*}\right))dt+\\ & & (u_{2}^{s}\cdot \nabla u_{2}^{s}\left(t^{*}\right)-u_{1}^{s}\cdot \nabla u_{1}^{s}\left(t^{*}\right))dt\\ & & +O(dt^{2}) \end{array}$$ (6)

The pair diffusivity at time t* is, K* = 〈l* · **v**(l*)〉—we ignore constants of proportionality, like 2, because we are interested only in the power scalings in this work. The KS equivalent is $K^{s*}=\langle l^{s*}\cdot \tilde{v}\left(l^{s*}\right)\rangle$. Using Eqs (5) and (6) and ignoring terms of order *dt*^2^ and higher,

$$\begin{array}{ccc} K^{s*} & \approx & \left\langle l^{*}\cdot v\left(l^{*}\right)\right\rangle +\left\langle l^{*}\cdot v^{s}\left(l^{*}\right)\right\rangle +\\ & & \langle l^{*}\cdot (u_{2}^{s}\left(t\right)\cdot \nabla u_{2}\left(t^{*}\right)-u_{1}^{s}\left(t\right)\cdot \nabla u_{1}\left(t^{*}\right))\rangle dt+\\ & & \langle l^{*}\cdot (u_{2}^{s}\left(t\right)\cdot \nabla u_{2}^{s}\left(t^{*}\right)-u_{1}^{s}\left(t\right)\cdot \nabla u_{1}^{s}\left(t^{*}\right))\rangle dt+\\ & & \left\langle v^{s}\left(l\right)\cdot v\left(l^{*}\right)\right\rangle dt+\left\langle v^{s}\left(l\right)\cdot v^{s}\left(l^{*}\right)\right\rangle dt \end{array}$$ (7)

The incremental timestep error between the KS and physical diffusivities for a given timestep *dt* is, *E*_*K*_ = |*K*^s^* − K*|. Using the expansion $u^{s}\left(x_{2}\left(t\right)\right)\approx u_{1}^{s}+l\cdot \nabla u_{1}^{s}$ in Eq (7), yields

$$\begin{array}{ccc} E_{K} & \approx & \langle l^{*}\cdot v^{s}\left(l^{*}\right)\rangle +\\ & & \langle l^{*}\cdot \left(u_{1}^{s}\cdot \nabla \right)v\left(l^{*}\right)\rangle dt+\\ & & \langle l^{*}\cdot \left(u_{1}^{s}\cdot \nabla \right)v^{s}\left(l^{*}\right)\rangle dt+\\ & & \langle l^{*}\cdot \left(l\cdot \nabla \right)u_{1}^{s}\cdot \nabla u_{2}\left(t^{*}\right)\rangle dt+\\ & & \langle l^{*}\cdot \left(l\cdot \nabla \right)u_{1}^{s}\cdot \nabla u_{2}^{s}\left(t^{*}\right)\rangle dt+\\ & & \langle v^{s}\left(l\right)\cdot v\left(l^{*}\right)\rangle dt+\\ & & \langle v^{s}\left(l\right)\cdot v^{s}\left(l^{*}\right)\rangle dt \end{array}$$ (8)

The time scale of the sweeping *T*_*s*_ is much larger than the local time scale of the pair separation. Hence, in the last four terms **l**(*t*) is replaced by **l**(t*) without affecting their magnitudes or scalings (the associated errors are ∼*O*(*dt*^2^) which is neglected).

All the terms in Eq (8) are now evaluated at the same time t*, so without loss of generality t* is replaced by *t* and the superscript ‘*’ is dropped. The subscript ‘_1_’ is also dropped because of homogeneity. Eq (8) now simplifies to,

$$\begin{array}{ccc} E_{K} & \approx & \langle l\cdot v^{s}\rangle +\\ & & \langle l\cdot \left(u^{s}\cdot \nabla \right)v\rangle dt+\\ & & \langle l\cdot \left(u^{s}\cdot \nabla \right)v^{s}\rangle dt+\\ & & \langle l\cdot \left(l\cdot \nabla \right)u^{s}\cdot \nabla u_{2}\rangle dt+\\ & & \langle l\cdot \left(l\cdot \nabla \right)u^{s}\cdot \nabla u_{2}^{s}\rangle dt+\\ & & \langle v^{s}\cdot v\rangle dt+\\ & & \langle {\left(v^{s}\right)}^{2}\rangle dt \end{array}$$ (9)

It is reasonable to assume that the (residual) sweeping flow field **u**^*s*^, which is caused by the largest of the inertial range eddies, is close to uniform across small distances, and therefore the relative velocities across the local separation scales *l* that it induces is small, i.e. gradients like |(**l** · ∇)**u**^*s*^| ≈ 0. However, gradients of the relative velocity **v**(*l*) itself can be large. The magnitude of *u*^*s*^ = |**u**^*s*^| is assumed large compared to *v*(*l*) = |**v**(*l*)|, and also as compared to *v*^*s*^(*l*) = |**v**^*s*^(*l*)|. *u*^*s*^ scales differently to *v*(*l*).

*v*(*l*) also scales differently to *v*^*s*^(*l*), the former being governed by inertial range turbulence scaling, and the latter by *differences* in the residual sweeping velocity across a small distance *l*. This can be seen clearly in the limit of uniform (parallel) sweeping flow, where the **v**(*l*) is unaffected, but **v**^*s*^(*l*) = **0** and all the terms on the right hand side in Eq (9) are zero or nearly zero compared to the second term. This indicates that the second term in Eq (9) makes the dominant contribution to the error.

Consider generalized energy spectra of the form *E*(*k*) = *ε*^2/3^*L*^5/3−*p*^*k*^−*p*^, for *k*_1_ ≤ *k* ≤ *k*_*η*_ and for 1 < *p* ≤ 3, and with *k*_*η*_/*k*_1_ ≫ 1 [9, 15]. In the swept frame of reference, *E*(*k*) = 0 for *k* < *k*_1_. The rate of energy dissipation is *ε* ∼ *U*^3^/*L*, where *U* is the velocity scale in the energy containing scales.

The previous discussion implies that $|{\nabla v}^{s}|\ll (\frac{U}{L})$ and therefore,

$$\begin{array}{c} v^{s}\left(l\right)\ll (\frac{l}{L})\;U. \end{array}$$ (10)

The energy in turbulent inertial scales local to *l* is, *v*^2^(*l*) ∼ *E*(1/*l*)/*l*, and therefore,

$$\begin{array}{c} v\left(l\right)∼{\left(\frac{l}{L}\right)}^{\frac{p-1}{2}}\;U \end{array}$$ (11)

$$\begin{array}{c} \text{and},\;\;|\nabla v\left(l\right)|∼{\left(\frac{l}{L}\right)}^{\frac{p-3}{2}}\;\frac{U}{L} \end{array}$$ (12)

It is usual to assume the scaling *l* ∼ *σ*_*l*_ as previously mentioned. Then, the second term in Eq (9) is given by,

$$\begin{array}{ccc} E_{2} & = & \left\langle l\cdot \left(u^{s}\cdot \nabla \right)v\right\rangle dt∼{\left(\frac{\sigma_{l}}{L}\right)}^{\frac{p-1}{2}}\;Uu^{s}\left(l\right)dt, \end{array}$$ (13)

All the other terms in Eq (9), labeled respectively *E*_1_, *E*_3_, …, *E*_7_, scale proportional to *u*^*s*^ or are much smaller, and this leads to the following estimates,

$$\begin{array}{ccc} \frac{E_{1}}{E_{2}} & \ll & {\left(\frac{\sigma_{l}}{L}\right)}^{\frac{5-p}{2}}\;\frac{L}{Udt},\;\\ \frac{E_{3}}{E_{2}} & \ll & {\left(\frac{\sigma_{l}}{L}\right)}^{\frac{3-p}{2}}\;\\ \frac{E_{4}}{E_{2}} & \ll & {\left(\frac{\sigma_{l}}{L}\right)}^{\frac{5-p}{2}}\;\\ \frac{E_{5}}{E_{2}} & \ll & {\left(\frac{\sigma_{l}}{L}\right)}^{\frac{5-p}{2}}\;\\ \frac{E_{6}}{E_{2}} & \ll & {\left(\frac{\sigma_{l}}{L}\right)}^{1}\;\\ \frac{E_{7}}{E_{2}} & \ll & {\left(\frac{\sigma_{l}}{L}\right)}^{\frac{5-p}{2}}. \end{array}$$ (14)

All of these ratios are small for *σ*_*l*_/*L* < 1 and 1 < *p* ≤ 3. This is true even in the expression for *E*_1_/*E*_2_ because the factor *L*/*Udt* is subdued by the very small factor in the brackets. It is reasonable to conclude that the 2nd term in Eq (9) is dominant and therefore *E*_*K*_ ≈ *E*_2_.

To estimate *E*_2_ itself, an estimate for *u*^*s*^(*l*) is needed. The inertial subrange contains only a small part of the total turbulence energy across the entire wavenumber range, 0 < *k* < ∞; a typical turbulence spectrum is the von Karman spectrum *E*_*vk*_(*k*). The von Karman spectrum is essentially a fit between the low wavenumber energy spectrum (*E*(*k*) ∼ *k*^4^), and the high wavenumber inertial subrange spectrum (*E*(*k*) ∼ *k*^−5/3^); the exact form is not important for our purposes; but see [16].

Let, the fraction of the turbulence energy in the inertial part of the spectrum be, *I*^2^ = *E*_*IN*_/*E*_*TOT*_, where *E*_*IN*_ is the energy in the inertial range, and *E*_*TOT*_ is the total turbulence energy. Then if, $E_{IN}∼u_{ks}^{2},$ and *E*_*TOT*_ ∼ *U*^2^, then we obtain, $I^{2}∼u_{ks}^{2}/U^{2}$, and *u*_*ks*_ ≈ *IU*. The choice of wavenumber which separates the inertial range from the large scales is somewhat arbitrary.

The wavenumbers that contribute to the sweeping of particle pairs at separation *σ*_*l*_ are in the range *k*_1_ ≤ *k* < *k*_*l*_, where *k*_*l*_ ∼ 1/*σ*_*l*_. In the KS sweeping frame of reference, the larger inertial scales are the sweeping scales, and the energy in these scales is approximately,

$$\begin{array}{ccc} {\left(u^{s}\right)}^{2} & ∼ & \int_{k_{l}}^{k_{1}}\;E\left(k\right)dk=\int_{k_{l}}^{k_{1}}\;\alpha_{k}\varepsilon^{2/3}L^{5/3-p}k^{-p}dk \end{array}$$ (15)

and using *ε* ∼ (*u*_*ks*_)^3^/*L* ∼ (*IU*)^3^/*L*, this gives

$$\begin{array}{ccc} u^{s} & \approx & IU\sqrt{1-{\left(\frac{\sigma_{l}}{L}\right)}^{p-1}} \end{array}$$ (16)

Using this in Eq (13), the residual error between the physical and the KS pair diffusivities in the inertial subrange of pair separations in the swept frame of reference, per unit timestep (retaining *E*_*K*_ to represent this quantity), is

$$\begin{array}{ccc} E_{K}\left(p\right)\approx E_{2} & = & C_{k}\sqrt{1-{\left(\frac{\sigma_{l}}{L}\right)}^{p-1}}\;{\left(\frac{\sigma_{l}}{L}\right)}^{\frac{p-1}{2}}\;U^{2}I. \end{array}$$ (17)

*C*_*k*_ is the constant of proportionality, which can depend upon *p*.

For *p* = 5/3, the residual error per unit timestep is,

$$\begin{array}{ccc} E_{K}\left(5/3\right) & \approx & C_{k}\sqrt{1-{\left(\frac{\sigma_{l}}{L}\right)}^{2/3}}\;{\left(\frac{\sigma_{l}}{L}\right)}^{1/3}\;U^{2}I. \end{array}$$ (18)

As *p* → 1, *E*_*K*_ → *E*_*K*_(1) ≈ *C*_*k*_*U*^2^/*L* ≈ *Constant* for *σ*_*l*_/*L* < 1, but is nearly zero close to *σ*_*l*_/*L* = 1. In this limit, *p* → 1, the pair diffusion is strongly local and is not affected by long range sweeping.

For *p* = 3, the residual error per unit timestep is negligibly small for *σ*_*l*_/*L* < 1,

$$\begin{array}{ccc} E_{K}\left(3\right) & \approx & C_{k}\sqrt{1-{\left(\frac{\sigma_{l}}{L}\right)}^{2}}\;(\frac{\sigma_{l}}{L})\;U^{2}I\ll E_{k}\left(5/3\right). \end{array}$$ (19)

In this limit, nearly all the energy is contained in the largest scales and inertial subrange scaling is no longer applicable.

### 2.2 The integrated error

The incremental timestep error in the diffusivity *E*_*K*_ only tells us the error in KS due to the lack of dynamic sweeping per numerical time step. As the particle pair separations increase (on average), these errors will not only increase, but they will also accumulate over a period of time. The important question is, does this integrated error remain small?

We therefore need to calculate the integrated error, relative to the actual diffusivity, over the period while the pair separation remains within the inertial subrange. The limit that the pair separation approaches the upper end of the inertial subrange, *σ*_*l*_ ∼ *L*, implies that *σ*_*l*_/*η* ≫ 1. In the limit of infinite inertial subrange this corresponds to *σ*_*l*_/*η* → ∞.

**Fig 2:**
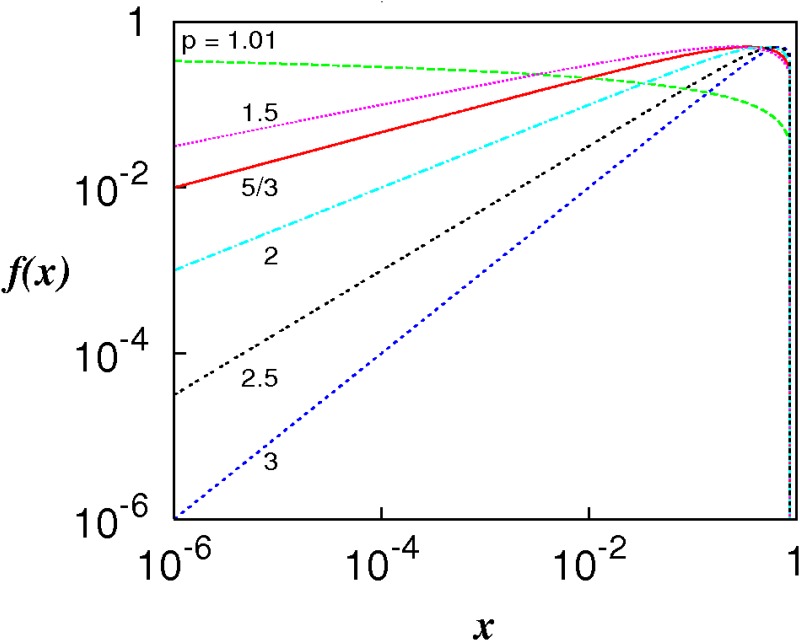
The function $f\left(x\right)=\sqrt{1-x^{p-1}}x^{(p-1)/2}$, 0 < *x* ≤ 1, for selected powers 1 < *p* ≤ 3.

Eq (17) provides a way of estimating an upper bound for the integrated residual error. Fig 2 shows the log-log plots of the factor $f\left(x\right)=\sqrt{1-x^{p-1}}x^{(p-1)/2}$ for selected powers in the range 0 < *p* ≤ 3. The range over which $\sqrt{1-x^{p-1}}\approx 0$ is very short and close to *x* = 1; but over the rest of the range $\sqrt{1-x^{p-1}}\approx 1$. Hence, to a good approximation, *f*(*x*) < *x*^(*p*−1)/2^ for all *x* ≤ 1, and using this in Eq (17) with *x* = *σ*_*l*_/*L* yields,

$$\begin{array}{ccc} E_{K}\left(p\right) & < & C_{k}U^{2}I\;{\left(\frac{\sigma_{l}}{L}\right)}^{\frac{p-1}{2}}. \end{array}$$ (20)

The integrated residual error, $E_{K}^{I}$, over a period of time is,

$$\begin{array}{c} E_{K}^{I}<C_{k}U^{2}I\;\int_{0}^{t}\;{\left(\frac{\sigma_{l}}{L}\right)}^{\frac{p-1}{2}}\;dt. \end{array}$$ (21)

Assuming the pair separation scaling $\sigma_{l}^{2}∼t^{\chi_{p}}$, where *t* is the time and for some *χ*_*p*_ > 0, yields,

$$\begin{array}{c} E_{K}^{I}≲C_{k}UIL\;{\left(\frac{\sigma_{l}}{L}\right)}^{\frac{p-1}{2}+\frac{2}{\chi_{p}}}. \end{array}$$ (22)

If the pair diffusivity scales like, $K∼\sigma_{l}^{\gamma_{p}}$, for some *γ*_*p*_ > 0 then *χ*_*p*_ = 1/(1 − *γ*_*p*_/2) is an exact relation.

The most important quantity is the relative integrated residual error with respect to the pair diffusivity, $e_{K}^{I}$. Using the above expression for *χ*_*p*_, and replacing the scaling with *L* by scaling with *η*, leads to

$$\begin{array}{c} e_{K}^{I}=\frac{E_{K}^{I}}{K}≲\frac{C_{k}UIL}{{\left(\frac{\sigma_{l}}{\eta }\right)}^{2\gamma_{p}-\frac{p+3}{2}}}. \end{array}$$ (23)

For strict locality scaling *γ*_*p*_ = (1 + *p*)/2, and this becomes

$$\begin{array}{c} e_{K}^{I}≲\frac{C_{k}UIL}{{\left(\frac{\sigma_{l}}{\eta }\right)}^{\gamma_{p}-1}}. \end{array}$$ (24)

Since *γ*_*p*_ − 1 > 0, $e_{K}^{I}$ decreases with increasing pair separation for all *p* > 1. For *p* = 5/3, we have *γ*_*p*_ = 4/3 and we obtain,

$$\begin{array}{c} e_{K}^{I}≲\frac{C_{k}UIL}{{\left(\frac{\sigma_{l}}{\eta }\right)}^{1/3}}. \end{array}$$ (25)

Thus, as *σ*_*l*_/*η* → ∞ then $e_{K}^{I}$ decreases; and as *σ*_*l*_/*η* → 0 then $e_{K}^{I}$ increases.

Even for non-local scaling, assuming that *γ*_*p*_ does not deviate too far from the local scaling, the above order of magnitude for $e_{K}^{I}$ is still approximately true. In fact, in KS we know, Section 3.3, that *γ*_*p*_ is slightly greater than locality so the errors will be slightly smaller than in Eq (25).

However, between these two asymptotic limits there must exist an intermediate range of separations, between 1 < *σ*_*l*_/*η* < ∞, where the errors remain negligible. In reality, all turbulence spectra are finite in range. So what size of the inertial subrange do we need to approximate an ‘infinite’ subrange?

Even then, the crucial question is: is the intermediate range of scales where the KS sweeping errors are negligible wide enough for inertial range scaling to be actually observable in KS?

To determine the extent of this intermediate range of scales, if it exists at all, simulations with KS must be performed with very large inertial subranges.

The errors in Eqs (24) and (25) cannot be determined directly from simulations because only the KS particle trajectories are generated directly.

However, the effects of the error analysis can be observed in several important ways that will be described in the next section where we carry out KS calculations for the pair diffusivity.

## 3 Simulations and results

The normalised integrated error, $e_{K}^{I}$, is scale dependent and reduces with increasing separation. The KS diffusivity is given by, $K^{s}\approx K\left(1+e_{K}^{I}\right)\to K$ as *σ*_*l*_/*η* → ∞. It is expected that if there is an appreciable intermediate range where the errors are negligible, then the power scaling in *K*^*s*^ must be constant and asymptotic to the limiting case where *σ*_*l*_/*η* → ∞. The extent of this intermediate range is determined by the range over which the power scaling in *K*^*s*^ is constant.

Furthermore, significant levels of the sweeping error means that the fluid particles cut through KS eddies, and therefore must be accompied by high levels of noise—the larger the relative sweeping error the larger the noise level.

Thus, where the errors are negligible it is expected that the correct power law scaling, *K*^*s*^ ≈ *K*, will be observed in that part of the of the inertial subrange.

On the other hand, where the errors are significant it is expected that *K*^*s*^ will deviate from the true power law scaling for *K* and also be accompanied with significant statistical noise due to fluid particles being swept through local eddies in that part of the inertial subrange. Even in this case, however, it is expected that the errors and the associated noise diminish as the pair separation increases.

### 3.1 Frames of reference

Comparison will be made between two cases: first, where *K*^*s*^ is obtained from KS in the physically correct sweeping frame of reference; and second, the case where large scale random sweeping velocities are explicitly added to the flow.

Case 1 **Swept frame.** Set the spectrum to be *E*(*k*) ∼ *k*^−*p*^ in the inertial subrange, and set *E*(*k*) = 0 for *k* < *k*_1_.
Case 2 **Non-swept frame.** Set the spectrum to be *E*(*k*) ∼ *k*^−*p*^ in the inertial subrange as in Case 1, and add *E*(*k*) = *E*_0_*δ*(*k* − *k*_0_) at some low wavenumber *k*_0_ < *k*_1_, and such that *E*_0_ is the energy in the von Karman spectrum in the range 0 < *k* < *k*_1_.

A very small fixed timestep, smaller than any timescale in the system *dt* ≪ *τ*_*η*_, is used in all the simulations reported here. *τ*_*η*_ is the Kolmogorov time scale.

The analysis for Case 2 is similar to that which leads to Eq (24), except that *U*, *L*, and *I* are different. From *I*^2^ ∼ *E*_*IN*_/*U*^2^, we obtain $UI∼\sqrt{E_{IN}}∼u_{ks}$ which remains relatively constant in the expressions (24) and (25). Thus, the differences in the errors between the two cases depends largely upon the different length scale *L* in the two cases, and assuming *A*_*k*_ = *C*_*k*_ *UI* ≈ *constant* we obtain,

$$\begin{array}{c} e_{K}^{I}≲A_{k}\;{\left(\frac{\sigma_{l}}{\eta }\right)}^{-\gamma_{p}+1}\;L. \end{array}$$ (26)

In Case 2, when large random scales are included in the simulations then *L* is about 10 times bigger than in Case 1. We may thereore expect the errors to be about 10 times bigger in Case2 than in Case 1.

The exact level of the error in Eq (26) depends upon the choice of cut-off scale between the sweeping and non-sweeping scales which is somewhat arbitray. It is best to view Eqs (24)–(26) as a general scaling law with parameters *U*, *L*, *I*, and the actual level of error can only be determined by simulations.

### 3.2 Kinematic Simulations

Kinematic Simulation [4, 5] is a Lagrangian method for particle diffusion in which the velocity fileld is prescribed as a sum of energy-weighted Fourier modes. It is akin to the widely used random flight type of statistical models in which the dynamical interactions between turbulent length scales is not explcitly modeled, rather the overall effect on the statistical moments of particle diffusion is mimicked. In KS this is accomplished by specifying the energy spectrum *E*(*k*). Although KS cannot capture the full dynamics of particle motion, it has the advantage that it can generate extremely large energy spectra that is far beyond current experimental or DNS capabilities, and therefore it can be used to examine some aspects of very high Reynolds number scaling laws. KS continues to be used in turbulent diffusion studies for both passive and inertial particle motion, including cases with generalized power-law energy spectra of the form *E*(*k*) ∼ *k*^−*p*^ for *p* > 1, Maxey [17], Turfus [18], Fung & Vassilicos [19], Malik & Vassilicos [20], Farhan et al. [21]. Meneguz & Reeks [22] carried out a DNS of inertial particle motion, and compared it to results from KS which they found to agree well with the DNS. Murray et al. [23] investigated inertial particle statistics using KS.

KS generates turbulent-like non-Markovian particle trajectories by releasing particles in flow fields that are incompressible by construction and which satisfy Eulerian statistics up to second order. A turbulent flow field realization is produced as a truncated Fourier series,

$$\begin{array}{c} W\left(x,t\right)=\sum_{n=1}^{N}\;[\left(A_{n}\times {\hat{k}}_{n}\right)\cos\left(k_{n}\cdot x+\omega_{n}t\right)+\left(B_{n}\times {\hat{k}}_{n}\right)\sin\left(k_{n}\cdot x+\omega_{n}t\right)]\;\;\;\; \end{array}$$ (27)

where *N* is a suitable number of representative wavemodes, typically hundreds for very long spectral ranges, *k*_*η*_/*k*_1_ ≫ 1. ${\hat{k}}_{n}$ is a random unit vector ($k_{n}={\hat{k}}_{n}k_{n}$ and *k*_*n*_ = |**k**_*n*_|). The coefficients **A**_*n*_ and **B**_*n*_ are chosen such that their orientations are randomly distributed and uncorrelated with any other Fourier coefficient or wavenumber, and their amplitudes are determined by $\langle A_{n}^{2}\rangle =\langle B_{n}^{2}\rangle \propto k_{n}E\left(k_{n}\right)$, where *E*(*k*), *k*_1_ ≤ *k* ≤ *k*_*η*_, is the turbulent energy specturm. The angled brackets 〈⋅〉 denotes the ensemble average over many flow fields. This construction ensures incompressibility in each flow realization, ∇ · **u** = 0. The flow field ensemble generated in this manner is statistically homogeneous, isotropic, and stationary.

An important feature of KS is that unlike some other Lagrangian methods, by generating entire kinematic flow fields in which particles are tracked it does not suffer from the crossing-trajectories error which is caused when two fluid particles occupy the same location at the same time in violation of incompressibility; but because KS flow fields are incompressible by construction this error is completely eliminated.

The energy spectrum *E*(*k*) can be chosen freely within a finite range of scales. In turbulent particle pair studies the interest is in Kolmogorov-like power law spectra,

$$\begin{array}{ccc} E(k) & = & C_{E}\varepsilon^{2/3}L^{5/3-p}k^{-p},\;\;k_{1}\leq k\leq k_{\eta }\left(=2\pi /\eta \right),\;\;1<p\leq 3 \end{array}$$ (28)

*C*_*E*_ is a constant. The largest represented scale of turbulence is 2*π*/*k*_1_, and the smallest is the Kolmogorov scale *η* = 2*π*/*k*_*η*_. The constant is normalized such that the total energy contained in the range *k*_1_ ≤ *k* ≤ *k*_*η*_ is 3(*u*′)^2^/2, where *u*′ is the rms turbulent velocity fluctuations in each direction. *ε*(*p*) is determined by integrating the spectrum, $\int_{k_{1}}^{k_{\eta }}E\left(k\right)dk=3{\left(u^{\prime }\right)}^{2}/2$. (*p* = 1 is a singular limit which is not consider here.) *v*_*η*_ = (*εη*)^1/3^ is the velocity micrcoscale, and *τ*_*η*_ = *ε*^−1/3^*η*^2/3^ is the Kolmogorov time micrcoscale.

The frequencies are chosen according to usual practice to be proportional to the eddy-turnover frequencies, i.e. $\omega_{n}=\lambda \sqrt{k_{n}^{3}E\left(k_{n}\right)}$. The choice of λ is somewhat arbitrary, but provided λ < 1 it does not affect the diffusion scaling itself—even frozen field with λ = 0 yields the same scaling [15]. λ = 0.5 is a common practice in KS which is also chosen here.

The distrbution of the wavemodes is geometric, *k*_*n*_ = *k*_1_*r*^*n*−1^, with *r* = (*k*_*η*_/*k*_1_)^1/(*N*−1)^. The grid size in wavemode-space of the *n*^*th*^ wavemode is $\delta k_{n}=k_{n}\left(\sqrt{r}-1/\sqrt{r}\right)$.

A particle trajectory is obtained by integrating the Lagrangian velocity **W**_*L*_(*t*),

$$\begin{array}{c} \frac{dx}{dt}=W_{L}\left(t\right)=W\left(x,t\right). \end{array}$$ (29)

The method of computing trajectories in KS is well established in the literature, see previous references above. Briefly, since an individual KS flow field realization is smooth and differentiable, Eq (29) can be readily integrated using any standard method such as Runga-Kutta or Predictor-corrector methods. The time step Δ*t* should be smaller than any other time scale in the system—in this case the Kolmogorov time scale *τ*_*k*_ = 2*π*/*k*_*η*_; thus we require Δ*t* ≪ *τ*_*k*_. In our simulations, particle trajectories are produced by integrating Eq (29) with a fixed time step of Δ*t* ≈ 0.01*τ*_*k*_ in a fourth order Adams-Bashforth predictor-corrector method.

It is important to produce a very large ensemble of independent particle pair trajectories. Only eight independent particle pairs are released in any one given KS flow realization, each pair set part by more than an integral length scale. Each pair is initially released with a pair separation distance of *l*_0_/*η* = 0.5. To obtain a true ensemble, this process is repeated in many thousands of KS flow realizations. (Releasing thousands of particle pairs in the same KS flow field will not produce the required independence which may produce biased statistics.) Pairs of trajectories are thus harvested over a large ensemble of flow realizations and pair statistics are then obtained from it for analysis.

The turbulent diffusivity itself can be computed in two ways. Directly from the forumla *K*(*l*) ∼ 〈**l** · **v**(*l*)〉, i.e. the ensemble average of the scalar producted of **v** and **l**. But it has been found that using the equivalent formula, *K*(*l*) ∼ *d*〈*l*^2^〉/*dt*, i.e. the derivative of the 〈*l*^2^〉, converges faster statistically needing a much smaller ensemble of trajectories, although the two methods give identical results for large enesmbles of particle trajectories. The latter method has been adopted here.

Lagrangian statistics are the physically meaningful output from KS. It is *not* correct to compare the kinematically generated flow fields directly with DNS flow fields. As such, KS is like Lagrangian methods such as Random Walk models where an individual particle trajectory has no physical meaning, but the ensemble average over many such random trajectories produces physically meaningful Lagrangian statistics.

### 3.3 Results

KS simulations were performed with *L* = 1, *k*_1_ = 1/*L* = 1, and *k*_*η*_ = 10^6^, *C*_*E*_ = 1.5 (Kolmogorov constant) and *u*′ = 1. There were 200 wavemodes per realization.

In Case 1 (swept frame of reference), *E*(*k*) = 0 for *k* < 1.

In Case 2, large scale random sweeping were added at the low wavenumber *k*_0_ = 1/10, with *E*(*k*) = *E*_0_*δ*(*k* − *k*_0_). The energy in these sweeping scales, *E*_0_, was equal to the energy contained in the von Karman turbulence spectrum for *k* < 1. *k*_0_ corresponds approximately to the location of the peak in the von Karman spectrum.

In both cases, three different power spectra were considered with, respectively, *p* = 1.01, 5/3, and 3. With 8 pairs released in 5000 flow realizations, the Lagrangian statistics were obtained from 40,000 particle pair trajectories.

**Fig 3:**
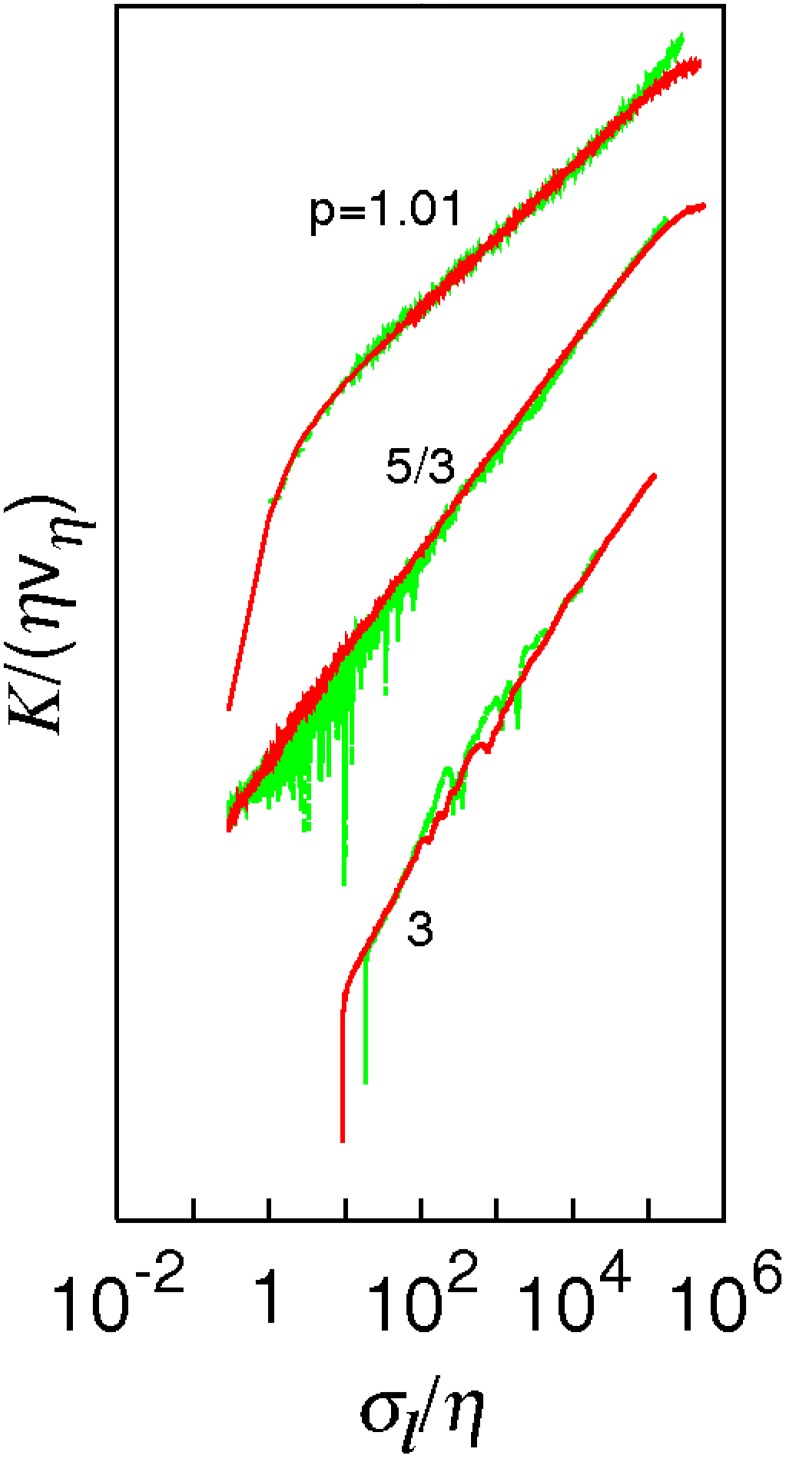
The turbulent diffusivity as log(*K*/(*ηv*_*η*_)) against log(*σ*_*l*_/*η*) obtained from KS. From top to bottom, *p* = 1.01, 5/3, 3. Case 1 (red lines), Case 2 (green lines). No scale is show on the vertical axis because the energies in the two cases are different and Case 2 plots have been shifted vertically in order to compare the two cases directly. This does not affect the scalings (the slopes).

Fig 3 shows log-log plots of the pair diffusivity *K*/(*ηv*_*η*_) against *σ*_*l*_/*η*, for Case 1 (red lines) and Case 2 (green lines). The energies in the two cases are different, so Case 2 plots have been shifted vertically in order to compare the two cases directly. This does not affect the scalings (the slopes) which is the main interest here. Hence the ordinate is shown without scale.

For *p* = 1.01, the two cases align with a constant power-law scaling, *γ*_1.01_ ≈ 1.07, over most of the inertial subrange of scales and there is very little statistical noise, indicating that $e_{K}^{I}\left(\sigma_{l}\right)\ll 1$ at all separations in this part of the inertial subrange in both cases. In this limit, locality is very strong, and the relative motion is unaffected by the long range sweeping. The obtained slope is indeed very close to the exact locality scaling of 1.005.

For *p* = 5/3 (Kolmogorov turbulence), in Case 1 (red) a clear power-law scaling is observed, *γ*_*Kol*_ ≈ 1.53 > 4/3, and very little statistical noise in the range 1 < *σ*_*l*_/*η* < 10^5^, indicating that $e_{K}^{I}\left(\sigma_{l}\right)\ll 1$ in this range of scales. Case 2 (green) deviates increasingly from Case 1 at small inertial separations where it is also accompanied with increasing levels of noise. Nevertheless, the agreement between the two cases for *σ*_*l*_/*η* > 10^2^ is good.

For *p* = 3, the two cases overlap with a power-law scaling, *γ*_3_ = 2, with almost no statistical noise. In this limit nearly all the energy is in the large scales and inertial range scaling is no longer applicable; rather uniform strained motion with the characteristic slope of 2 is obtained.

## 4 Discussion and conclusions

All the results in Fig 3 are consistent with the numerical analysis and the theoretical predictions in section 3. The constant power law scaling over most of the inertial subrange of separations, 1 < *σ*_*l*_/*η* < 10^5^, and the very low level of statistical noise in the swept frame of reference are especially important. (The departure from this for *σ*_*l*_/*η* < 1 observed in Fig 3 is outside of the inertial subrange.)

The KS sweeping error in this frame of reference is therefore negligible for most practical purposes. It is possible that KS could produce negligible sweeping errors in even bigger intermediate ranges than reported here, but the current simulations are the maximum size of inertial subrange possible, *k*_*η*_/*k*_1_ = 10^6^, with double-precision accuracy.

It is remarkable that even when large scale sweeping is included, the KS sweeping errors remain small in the range *σ*_*l*_/*η* > 10^2^.

It is also noted that some Direct Numerical Simulation (DNS) show pair diffusion which appear to display locality scaling, see [24] for example. However, the maximum inertial range obtained in DNS to date is around *k*_*η*_/*k*_1_ ≈ 10^2^, which is much shorter than required to test pair diffusion scaling reliably—that requires *k*_*η*_/*k*_1_ > 10^4^. Thus the current KS results cannot be compared directly with DNS at the present time. However, for low Reynolds KS has been validated against DNS for turbulent pair diffusion by Malik & Vassilicos [20]; here not only did the pair diffusion from KS closely match the DNS results with the same energy specturm, but the fourth order statistic, the kurtosis in the pair separation, also matched remarkably well.

The main contribution of this work is that it has been shown that the departure from locality scaling observed in KS cannot be attributed to the sweeping effect; in a reference frame moving with the large energy containing scales in zero mean flow turbulence the sweeping errors in the turbulent pair diffusion process in KS is negligible in the inertial subrange where, 1 < *σ*_*l*_/*η* < 10^5^.

But if the sweeping errors in KS are negligible, why is locality scaling not observed for *p* = 5/3 where KS yields *γ*_*Kol*_ ≈ 1.53 > 4/3—could the locality hypothesis itself be in error? This cannot be concluded for certain from the present results alone—only detailed experiments or DNS can provide a definitive answer to that, and both are decades away at the present time. But the results presented here, do excite an important new line of thinking that has been neglected in the turbulence community, but one which has as much validity per se as the locality hypothesis itself. A non-local theory of pair diffusion that could explain the observed results and available data is currently the subject of active research by the author.
